# Perioperative cognition in association with malnutrition and frailty: a narrative review

**DOI:** 10.3389/fnins.2023.1275201

**Published:** 2023-11-02

**Authors:** Vikalpa Dammavalam, Jasper Murphy, Meenu Johnkutty, Murad Elias, Ryan Corn, Sergio Bergese

**Affiliations:** ^1^Department of Neurology, Stony Brook University Hospital, Stony Brook, NY, United States; ^2^Renaissance School of Medicine, Stony Brook University, Stony Brook, NY, United States; ^3^Department of Anesthesiology, Stony Brook University Hospital, Stony Brook, NY, United States

**Keywords:** frailty, nutrition, prehabilitation, perioperative cognition, postoperative delirium

## Abstract

Postoperative delirium (POD) is a prevalent clinical entity characterized by reversible fluctuating altered mental status and cognitive impairment with acute and rapid onset a few days after major surgery. Postoperative cognitive decline (POCD) is a more permanent extension of POD characterized by prolonged global cognitive impairment for several months to years after surgery and anesthesia. Both syndromes have been shown to increase morbidity and mortality in postoperative patients making their multiple risk factors targets for optimization. In particular, nutrition imparts a significant and potentially reversible risk factor. Malnutrition and frailty have been linked as risk factors and predictive indicators for POD and less so for POCD. This review aims to outline the association between nutrition and perioperative cognitive outcomes as well as potential interventions such as prehabilitation.

## 1. Introduction

Postoperative delirium (POD) is a prevalent clinical entity characterized by reversible fluctuating altered mental status and cognitive impairment with acute and rapid onset a few days after major surgery. Other characteristics include hallucinations, inappropriate behavior and functional decline. Most notably, POD has relevant clinical implications of increased perioperative morbidity and mortality as well as increased healthcare costs (Saczynski et al., 2012; Bilotta et al., 2013; Alam et al., 2018; Anderson et al., 2018). Postoperative cognitive decline (POCD) is a more permanent extension of POD characterized by prolonged global cognitive impairment for several months to years after surgery and anesthesia. This syndrome is less defined and includes a range of impairments in memory, learning and concentration. Therefore, diagnosis often requires neuropsychological testing as these subtleties are less clinically apparent (Moller et al., 1998; Bilotta et al., 2013; Evered and Silbert, 2018; Yang et al., 2022). Implications are similar to POD with increased morbidity and mortality (Yang et al., 2022).

Etiology of POD and POCD remains unclear but is likely multifactorial. Some studies suggest upregulated systemic cytokine markers – specifically neuron specific enolase, glial fibrillary acidic, C-reactive protein and interleukin 6 – due to post-surgical neuroinflammation as the pathophysiology of delirium but no clear pathway has been identified (Alam et al., 2018; Anderson et al., 2018). However, multiple risk factors for POD and POCD have been identified in the three stages of surgery: preoperative, intraoperative and postoperative. Of all the potential risk factors, preoperative ones may be the ideal targets for optimization to reduce the risk of developing cognitive deficits. In particular, nutrition imparts a significant and potentially reversible risk factor. One significant consequence of malnutrition is its association with clinical frailty, a state of increased vulnerability to stressors due to reduced physiological reserves across multiple bodily systems (McIsaac et al., 2020). This review aims to outline the association between nutrition and perioperative cognitive outcomes as well as potential interventions.

### 1.1. Postoperative delirium risk factors

Preoperative risk factors include age, type of surgery, co-morbidities and metabolic changes. Demographics of older age, female sex and non-Caucasian race as well as socioeconomic features of lower education level are associated with development of POD (Saczynski et al., 2012). Age above 70 years, and especially above 80 years is a major risk factor associated with higher odds of POD although a systematic review showed many studies quoted mean ages in the 60s as well (Raats et al., 2016). Particular surgeries such as orthopedic, abdominal aortic aneurysm and cardiothoracic surgery are strong predictors of increased risk of POD (Bilotta et al., 2013). In fact, incidence of POD ranges broadly from up to 75% of patients who underwent cardiac surgery versus only 37% of patients who underwent lung transplant surgery (Anderson et al., 2018). In post coronary artery bypass grafting (CABG) patients, co-morbidities of obesity, hypertension, diabetes, peripheral vascular disease, atrial fibrillation, stroke and most importantly, pre-existing cognitive impairment conferred statistically significant risk of POD (Greaves et al., 2020). In all surgical patients, preoperative presence of cognitive impairment, neurodegenerative disease, cerebrovascular disease or psychiatric disease (especially depression) expectedly correlated with increased risk for POD, especially in intensive care unit (ICU) setting (Saczynski et al., 2012; Bilotta et al., 2013; Raats et al., 2016). A systematic review by Raats et al. (2016) named hypercholesterolemia and diabetes, among others, as independent risk factors for POD; both of which can be influenced positively with nutritional management. Obstructive sleep apnea is associated with higher rates of POD likely due to intermittent hypoxia. In addition to oxygen deprivation, metabolic abnormalities such as hyponatremia and low serum albumin confer increased risk of POD (Bilotta et al., 2013) which also supports the need for preoperative nutritional optimization.

Intraoperative risk factors are fewer and likely less impactable given the constraints of surgery and clinician preference. Pathologic factors negatively affecting intraoperative cerebral blood flow such as severe bleeding, hypotension and hypocapnia are significant risk factors for increased incidence of POD (Bilotta et al., 2013), especially in post lung transplant patients (Smith et al., 2016). Particular anesthetics such as ketamine and propofol are also associated with increased risk of POD (Bilotta et al., 2013). In contrast to guidelines for benzodiazepine use in the ICU in the elderly, a recent meta-analysis of 34 randomized controlled trials revealed perioperative benzodiazepine use may not increase risk of POD. Still, subgroup analysis showed 80% higher incidence of POD with benzodiazepines as compared to dexmedetomidine (Wang et al., 2023). Studies have shown preoperative anticholinergic load is an independent risk factor for development of POD (Mueller et al., 2020; Herrmann et al., 2022). In post-CABG patients, increased intubation time and duration of surgery are the two largest statistically significant risk factors (Greaves et al., 2020).

Postoperative risk factors are often co-associated with ICU related complications. Similar to preoperative risks, poor cardiac function, new onset atrial fibrillation and persistent hypoxia accompany increased chance of POD. Other clinical factors such as sleep deprivation, bladder catheterization and central venous catheterization also precipitated POD (Bilotta et al., 2013). Length of stay (LOS) in ICU was the strongest postoperative risk factor in post-CABG patients (Greaves et al., 2020).

### 1.2. Postoperative delirium outcomes

POD is linked to adverse outcomes such as increased LOS, morbidity and mortality.

In a prospective cohort study by Zhao et al., the impact of poor nutrition and LOS on POD in older patients undergoing non-cardiac surgery was investigated using the geriatric nutritional risk index (GNRI) within 48 h of hospital admission. This study determined a statistically significant association between level of nutritional risk and prolonged LOS of 14 days and concluded that patients with severe/moderate nutritional risk were more likely to develop POD (Zhao et al., 2020). Caccialanza et al. (2010) evaluated the nutritional status and LOS of 1,274 ambulatory adult patients admitted for medical or surgical treatment and similarly found that patients with worse nutritional status had increased LOS. Cereda et al. (2015) utilized the GNRI in a prospective multicenter hospital-based cohort study and also found increased nutritional risk was associated with prolonged LOS. With the available evidence in the literature, poor nutritional status is associated with increased LOS.

In addition to the increased morbidity and mortality associated with POD, there is also a negative impact on quality of life. In a prospective cohort study by Beishuizen et al., POD increased the odds substantially for functional decline and death in patients who underwent transcatheter aortic valve implantation (TAVI). Beishuizen et al. (2020) concluded that the risk of delirium is an important factor in the shared decision making prior to patients undergoing this procedure.

The association between frailty and increased postoperative mortality is well established in the literature (McIsaac et al., 2016, 2017; James et al., 2019). Frailty, which is a state of decreased functional reserve that is usually described as deficits in physical function as well as nutrition, cognition, and mental health, is often associated with advanced age. In a single-center, retrospective, observational study of 558 adults, Pedemonte et al. (2021) concluded that frailty is associated with increased postoperative mortality which may be mediated by POD in older orthopedic trauma patients. In general, POD is a relatively common complication, but one with serious consequences. According to Jin et al. (2020) POD can increase hospital stay by 3 days and has a 30-day mortality of upwards of 10%.

### 1.3. Postoperative cognitive decline risk factors

POD has expectedly been identified as a major risk factor for development of POCD in cardiac surgery (Saczynski et al., 2012) and likely other surgeries because they can be seen as temporally-associated entities on a spectrum of perioperative cognitive deficits.

Similar to preoperative risk factors in POD, older age above 60 (Monk et al., 2008), low education level and multiple comorbidities are independent risk factors for POCD in cardiac and noncardiac surgery (Moller et al., 1998; Saczynski et al., 2012; Yang et al., 2022). A major multicenter study, the International Study of Post-operative Cognitive Dysfunction, found the incidence of POCD in elderly patients to be 9.9% 3 months after surgery (Moller et al., 1998). A systematic review by Yang et al. (2022) found co-morbidities such as hypertension (Feinkohl et al., 2017), diabetes (Ozalp Horsanali et al., 2021) and depression (Bhushan et al., 2021) were particularly linked to POCD, likely due to minute cerebrovascular compromise. A meta-analysis by Travica found class III evidence of preoperative age and diabetes as risk factors in post-CABG patients (Travica et al., 2023). Chronic obstructive pulmonary disease is also a risk factor for POCD due to chronic hypoxia as seen with obstructive sleep apnea in POD (Bilotta et al., 2013). Type of surgery impacted incidence of POCD. CABG in particular yielded the highest increase in risk of POCD at 20 to 50% as compared to an incidence of 5 to 55% in noncardiac patients (Moller et al., 1998; Evered et al., 2011; Tasbihgou and Absalom, 2021; Yang et al., 2022; Travica et al., 2023). Nutritional status seems to be less of a factor in POCD than POD, however, a couple studies suggest vitamin D plays a role in development of POCD (Zhang et al., 2018; Hung et al., 2022).

As with all cognitive phenomena, intraoperative cerebral perfusion compromise and hypoxemia can increase risk of POCD. However, several studies showed development of POCD after cardiac and noncardiac surgeries even without cerebral perfusion compromise and hypoxemia suggesting age and preoperative comorbidities have a stronger influence (Moller et al., 1998; Evered and Silbert, 2018). In a narrative review by Kong et al. (2022) a side by side comparison of postoperative risk factors including length of ICU stay and need for pain medications appeared to be equivalent in POD and POCD.

### 1.4. Cognitive trajectories and outcomes

Cognitive trajectories varied significantly but in most studies, which evaluated cardiac surgical patients, trajectory was characterized by initial decline followed by prolonged impairment (Saczynski et al., 2012). In a prospective study of elderly patients without pre-existing dementia, Inouye et al. (2016) described a biphasic pattern with POD peaking at 1 month followed by full recovery at 2 months in the short term and a steep decline in the long term. Similarly, other studies quoted in Kong et al. (2022) narrative review showed near complete recovery of patients with delayed neurocognitive recovery defined as cognitive impairment within 30 days postoperative, however, patients with POCD defined as impairment beyond 30 days became indistinguishable from the generic neurocognitive disorders (NCD) at 12 months. This meshing of POCD into NCD was also described by Inouye et al. as a long term cognitive trajectory similar to that seen in mild cognitive impairment (Ely et al., 2001).

Monk et al. (2008) revealed a high incidence of 30–40% of postoperative patients who develop POCD on discharge, meaning the poor outcomes of POCD affect a significant portion of postoperative population. In general, patients with POCD have increased mortality after noncardiac surgery (Steinmetz et al., 2009). Monk et al. (2008) also described elderly patients over 60 with POCD on discharge and at 3 month had higher incidence of mortality within 1 year post operation. Quality of life is also impaired as Steinmetz et al. (2009) found higher rates of early retirement and more need for social and financial assistance.

## 2. Nutrition and postoperative cognition

### 2.1. Malnutrition

Malnutrition is multifaceted, carrying a large degree of physiological (Norman et al., 2021), sociological (Besora-Moreno et al., 2020), and psychological (Boulos et al., 2017) risk factors. With increasing age and a decline in social networks, the elderly are most vulnerable to malnutrition, making them a target population for preoperative nutritional interventions. In the preoperative setting, malnutrition depletes patients of the metabolic reserve required to return to baseline (Bañuls et al., 2019). Surgery is a major stressor that demands the body to respond adequately through a variety of mechanisms including an increase in hepatic glucose production (Imamura et al., 1975) and the catabolism and mobilization of amino acids into the blood stream (Souba et al., 1985). Without the appropriate physiological reserves to respond, those who are malnourished start the recovery process at a more vulnerable baseline (Kwag et al., 2014).

#### 2.1.1. Association of malnutrition and postoperative delirium

The etiology of delirium is multifaceted and has been linked to not only malnutrition, but also age (Gross et al., 2012), drugs (Clegg and Young, 2011), coexisting medical conditions, functional status (Inouye et al., 2014), preoperative cognitive status (Smith et al., 2009), among others.

Malnutrition poses a key risk factor for POD. In a cohort of 415 patients who underwent surgical repair for femur fractures, those identified as “overtly” malnourished were found to be 3.0 times more likely as those with normal nutritional status to develop POD. Malnourished participants were more likely to be disabled, cognitively impaired, and had higher in-hospital mortality than those at risk of malnutrition or who were well nourished (Mazzola et al., 2017). Similar associations between malnutrition and the incidence of POD have been observed in cardiac (Ringaitienė et al., 2015), spinal (Onuma et al., 2020), and colorectal surgery (Mosk et al., 2018) as well. Regardless of surgery type, malnutrition was an independent risk for the development of POD in the above cohorts.

Some explanations for the link between malnutrition and POD include an impairment in neurotransmission due to thiamine deficiency (Osiezagha et al., 2013; Moslemi et al., 2020) and the brain as uniquely vulnerable to malnutrition due to its increased metabolic demand. However, a clear mechanism has yet to be clearly delineated and remains to be further investigated.

Alcohol consumption is another nutritional factor that has been shown to be associated with postoperative delirium. A study of 252 patients found alcohol to be a risk factor for POD, particularly in quantities larger than 24 grams per day on average (Wu et al., 2023). Additionally, excessive alcohol intake can interfere with nutrient absorption and metabolism, affect dietary choices, and impair liver function, all of which may exacerbate malnourishment and increase risk for POD (Butts et al., 2023).

#### 2.1.2. Predictive tools for malnutrition and postoperative delirium

Given that malnutrition is an independent risk factor for POD, predictive tools for malnutrition may concurrently predict development of POD. Routinely, serum albumin is used as an indicator of nutritional status in the perioperative setting. However, its use is nonspecific and has been associated with other pathological states with increased inflammation (Ishida et al., 2014; Keller, 2019). To best assess malnutrition, preoperative screening assessments that incorporate the multidimensional nature of nutrition may be beneficial and prove to be more suitable in assessing risk for POD than serum albumin in isolation (Mazzola et al., 2017; Zhao et al., 2020).

One such assessment is the prognostic nutritional index (PNI) which reflects the immune-nutritional status by calculating the function of peripheral lymphocyte count and serum albumin. Hung et al. (2022) explored the association between PNI and POD in over 3,743 patients and found a nearly twofold increased risk of delirium compared to those with a relatively high PNI index. Other screening tools like the Mini Nutritional Assessment Short Form (MNA-SF) incorporates questions regarding food intake, neuropsychological conditions and mobility, among others, found similarly close associations between nutrition and POD. The MNA-SF has been well validated for use among the elderly (Guigoz, 2006; Kaiser et al., 2009).

Mazzola et al. (2017) observed a dose-effect relationship between lower scores on the MNA-SF and the incidence of POD. Similarly, lower scores on the MNA-SF were associated with the incidence of subsyndromal delirium as well, illustrating the MNA-SF’s sensitivity to capturing cognitive impairment just short of clinical delirium (Denny et al., 2020). Zhao et al. (2020) compared the use of the MNA-SF to the GNRI in predicting POD and LOS and found the GNRI used more objective data, requires less patient participation and is less time-intensive to calculate. Like the MNA-SF, the GNRI screening tool has also been validated for classification of nutritional status among the hospitalized elderly (Abd-El-Gawad et al., 2014). Cereda et al. (2009) demonstrated the use of the GNRI in predicting nutrition-related risk of death, with higher mortality associated with those identified at “severe risk” by the GNRI. While both the MNA-SF and the GNRI were independent predictors of prolonged LOS, the MNA-SF was deemed better at predicting development of POD (Ling et al., 2021). However, in those receiving total parenteral nutrition (TPN) or who had poor cognition at baseline, the use of the MNA-SF may be contraindicated, a limitation of this screening assessment (Zhao et al., 2020).

### 2.2. Frailty

Frailty is prevalent in the elderly population, ranging from 15% in the community to 54% among those hospitalized (Artaza-Artabe et al., 2016). Clinical frailty is often characterized by decreased muscle mass, strength, and overall physical function in addition to declining cognitive and psychological functioning. As the aging population in Western societies continues to grow at a rapid pace, there is a notable increase in the number of older individuals with frailty undergoing surgical procedures (McIsaac et al., 2020). These individuals, who are inherently vulnerable to stressors, face a heightened risk of experiencing adverse outcomes and requiring greater utilization of healthcare resources when exposed to the stress of surgery. One study found that frailty was associated with longer hospitalizations, more nonroutine discharges, and higher total hospital costs following spine surgery (Elsamadicy et al., 2022). Furthermore, emerging evidence suggests a strong association between clinical frailty and POD.

#### 2.2.1. Association of malnutrition and frailty

To define clinical frailty, several models have been created including the Fried Frailty phenotype. The Fried frailty phenotype is a widely recognized and validated operational definition of frailty developed by Fried et al. (2001). It identifies frailty based on five criteria: unintentional weight loss, self-reported exhaustion, low physical activity, slow gait speed, and weak grip strength. Individuals are considered frail if they meet three or more of these criteria (Fried et al., 2001). Inadequate nutritional status is one of the primary risk factors for the onset of frailty (Artaza-Artabe et al., 2016) and has been shown to impact all five criteria utilized in Fried’s Frailty phenotype (Bonnefoy et al., 2015). Other measures of frailty include Rockwood’s Frailty Index and the Clinical Frailty Scale, both of which also consider nutritional status in determining frailty.

Frailty is a multifactorial condition influenced by various factors, including age, physical activity levels, chronic diseases, and nutritional status. While there is no single specific nutritional component that causes frailty, deficiencies in certain nutrients can contribute to the development of frailty in older adults. Some of the key nutritional components that play a role in frailty include protein, vitamin D, calcium, omega-3 fatty acids, and antioxidants (O'Connell et al., 2020). Preoperative vitamin D deficiency in particular has been implicated in the development of POD and POCD (Zhang et al., 2018; Hung et al., 2022).

Studies have shown that the quality of the diet throughout one’s lifespan is related to the incidence of frailty and its co-occurrence with sarcopenia (Cruz-Jentoft et al., 2017). Consequently, targeted nutritional interventions hold promise in reducing the incidence of frailty and potentially reversing its progression.

#### 2.2.2. Association of preoperative frailty and postoperative delirium

The association between preoperative frailty and postoperative delirium has been extensively investigated in various experimental models, patient populations, and surgical procedures. Most studies utilized the five-item FRAIL scale, focusing on fatigue, resistance, ambulation, illness, and weight loss, along with the Confusion Assessment Method to quantify frailty and POD, respectively. Several studies used other validated metrics or in-house assessments. It is important to note that the assessments and tools used to screen and diagnose POD exhibit variability among studies, with some employing tools that have not been validated for this specific purpose. Screening and diagnosis of POD can be further complicated by variable frequency of POD assessments, subjectivity in diagnosis, interobserver variability, and the potential overlap of delirium symptoms with other medical conditions (Majewski et al., 2020). However, the relationship between preoperative frailty and POD has been observed in diverse surgical procedures, including spine surgery (Susano et al., 2020), elective brain surgery (Wang et al., 2021), orthopedic trauma (Esmaeeli et al., 2022), cardiac (Jung et al., 2015; Brown et al., 2016; Nomura et al., 2019), joint arthroplasty (Chen and Qin, 2021) and abdominal surgery (Tsai et al., 2022). Furthermore, meta-analyses conducted by Gracie, Fu, and Liu have consolidated and reinforced these findings with regards to surgery in general, providing compelling evidence for the link between preoperative frailty and POD (Gracie et al., 2021; Fu et al., 2022; Liu et al., 2022). This body of research underscores the importance of addressing frailty as a risk factor to minimize POD.

Thankfully, several of these experiments have shown the absence of long-term POCD associated with preoperative frailty (Nomura et al., 2019; Mahanna-Gabrielli et al., 2020). However, short-lasting POD remains a serious issue, impacting patient outcomes and healthcare costs (Elsamadicy et al., 2022). Efforts should be made to minimize its risk factors, including interventions such as nutritional supplementation.

## 3. Future directions

### 3.1. Reversal and prevention of frailty through proper nutrition

Given the strong association between inadequate nutrition and clinical frailty, nutritional supplementation is being considered for both prevention and treatment of frailty.

Three studies examined the relationship between protein intake and frailty in older adults. The Finnish study found that older women with higher protein intake experienced lower incidents of frailty (Isanejad et al., 2020). The European SHARE cohort study, which included both men and women aged 50 years and above, also showed a similar association between higher protein intake and reduced frailty (Haider et al., 2020). Additionally, the Newcastle 85+ study, which focused on very old adults, found a correlation between higher protein intake and decreased frailty (Mendonça et al., 2019). All three studies accounted for factors such as age, sex, and other potential confounders in their analyses.

Research regarding the reversal of existing frailty through nutritional supplementation has yielded varied findings. While several randomized controlled trials have shown that proper nutrition can indeed help reverse frailty and increase muscle mass indices (Ng et al., 2015; Park et al., 2018), a meta-analysis of eight studies by Oktaviana found that protein supplementation alone does not significantly improve muscle mass or frailty indices in the frail population (Oktaviana et al., 2020). However, more promising results have emerged when protein supplementation is coupled with exercise interventions. A meta-analysis of 22 randomized controlled trials revealed significant improvements in frailty status, lean mass, muscle strength, and physical mobility among frail older individuals (Liao et al., 2020). Further research is necessary to establish the precise parameters of effective interventions for the frail population, thus optimizing healthcare outcomes and enhancing overall patient well-being.

### 3.2. Prevention of malnutrition through Prehabilitation

Interventions designed to reduce postoperative complications may be beneficial by targeting modifiable risk factors like malnutrition through prehabilitation with nutritional supplementation as described above and exercises.

A single center study in the Netherlands investigated the use of multimodal prehabilitation to reduce the incidence of POD after elective abdominal surgery. A five-week period of prehabilitation, which included nutritional counseling and home-base exercises, reduced the incidence of POD by one third (Janssen et al., 2019). Another retrospective cohort study including over 160,151 patients investigated the use of early nutritional intervention on day 1 in malnourished patients undergoing hip/femur surgeries. While nutritional intervention was underutilized, when implemented it decreased LOS while not increasing hospital costs. Nutritional intervention did not improve secondary outcomes like infection, hospital mortality and ICU admission (Williams et al., 2021).

The use of cognitive activities during prehabilitation may also be considered in the reduction of POD, however, its role in reducing POD is still questionable. Humeidan et al. (2021) investigated the effect of patients completing 10 h of cognitive exercises preoperatively to the incidence of POD and concluded that patients who were at least minimally compliant with the cognitive training regimen had a decrease in POD incidence.

Implementing prehabilitation regiments designed to optimize a patient’s physical and cognitive well-being may be key to reducing postoperative complications like POD and implementation of such programs has shown promise for widespread use.

### 3.3. Conclusion

POD and POCD have various risk factors and contribute to higher rates of morbidity, mortality and poor quality of life in postoperative patients. Malnutrition and frailty have been linked as risk factors and predictive indicators for POD and less so for POCD. Preoperative prehabilitation methods combining nutritional supplementation, cognitive exercises and physical home exercises can aid in medical optimization of malnutrition and fragility to potentially reduce the risk of POD and POCD. Still, further trials investigating nutritional optimization are required to formulate a robust prehabilitation methodology.

## Author contributions

VD: Writing – original draft, Writing – review & editing. JM: Writing – original draft. MJ: Writing – original draft. ME: Writing – original draft. RC: Writing – review & editing. SB: Conceptualization, Writing – review & editing.
